# Rheumatoid arthritis increases the risk of malignant neoplasm of bone and articular cartilage: a two-sample bidirectional mendelian randomization study

**DOI:** 10.1186/s13075-023-03205-5

**Published:** 2023-11-13

**Authors:** Mingyi Yang, Yani Su, Ke Xu, Jiale Xie, Pengfei Wen, Lin Liu, Zhi Yang, Peng Xu

**Affiliations:** Department of Joint Surgery, HongHui Hospital, Xi’an Jiaotong University, Xi’an, 710054 Shaanxi China

**Keywords:** Rheumatoid arthritis, Neoplasm, Bone, Articular, Cartilage

## Abstract

**Objective:**

Prior research has revealed a heightened prevalence of neoplasms in individuals diagnosed with rheumatoid arthritis (RA). The primary objective of this study is to delve into the causal association between RA and two distinct types of neoplasms: benign neoplasm of bone and articular cartilage (BNBAC) and malignant neoplasm of bone and articular cartilage (MNBAC).

**Methods:**

We employed summary data from genome-wide association analyses (GWAS) to investigate the causal relationship between RA and two neoplasms, BNBAC and MNBAC, using a two-sample bidirectional Mendelian randomization (MR) study design. The IEU OpenGWAS database provided the GWAS summary data for RA, while the Finnish consortium supplied the GWAS summary data for BNBAC and MNBAC. Our analysis involved the utilization of eight distinct MR methods, namely random-effects inverse variance weighted (IVW), MR Egger, weighted median, simple mode, weighted mode, maximum likelihood, penalized weighted median, and fixed effects IVW. Subsequently, we conducted assessments to evaluate heterogeneity, horizontal pleiotropy, outliers, the impact of a single-nucleotide polymorphism (SNP), and adherence to the assumption of normal distribution in the MR analysis.

**Results:**

The results from the MR analysis revealed that there was no significant genetic association between RA and BNBAC (*P* = 0.427, odds ratio [OR] 95% confidence interval [CI] = 0.971 [0.904–1.044]). However, a positive genetic association was observed between RA and MNBAC (*P* = 0.001, OR 95% CI = 1.413 [1.144–1.745]). Conducting a reverse MR analysis, we found no evidence to support a genetic causality between BNBAC (*P* = 0.088, OR 95% CI = 1.041 [0.994–1.091]) or MNBAC (*P* = 0.168, OR 95% CI = 1.013 [0.995–1.031]) and RA. Our MR analysis demonstrated the absence of heterogeneity, horizontal pleiotropy, and outliers and confirmed that the effect was not driven by a single SNP. Additionally, the data exhibited a normal distribution.

**Conclusion:**

The findings of this study demonstrate that RA constitutes a significant risk factor for MNBAC. In the context of clinical application, it is advisable to conduct MNBAC screening in RA patients and remain vigilant regarding its potential manifestation. Importantly, the outcomes of this investigation introduce a fresh vantage point into the understanding of the tumorigenesis associated with RA.

**Supplementary Information:**

The online version contains supplementary material available at 10.1186/s13075-023-03205-5.

## Introduction

Rheumatoid arthritis (RA) is a chronic autoimmune inflammatory disease whose etiology remains unknown. It is characterized by inflammatory changes in joints, cartilage, and synovitis, with synovitis being a prominent feature of RA [1]. Clinically, RA presents as symmetrical, invasive joint inflammation affecting the small joints of the hands and feet, leading to pain, swelling, and joint stiffness. It predominantly affects women aged 30 to 50, with an incidence rate of 1 in 150 [2]. The disease progresses rapidly after onset, resulting in irreversible physical dysfunction and joint deformities. Growing evidence indicates that the development of RA is influenced by both genetic and environmental factors [3, 4]. Rheumatoid arthritis is a polygenic disorder with a substantial genetic component, estimated to have a heritability of 60% [4]. Recent genetic studies have identified more than 150 loci associated with RA, with the human leukocyte antigen (HLA) system exerting the strongest influence. HLA alleles have also been implicated in multiple interactions with environmental and other genetic risk factors, further increasing the susceptibility to RA [3]. Additionally, RA patients may exhibit extra-articular manifestations involving various organs, including interstitial lung disease, pericarditis, pleural effusion, or bronchiectasis. Importantly, RA patients have a higher risk of serious infections, respiratory diseases, osteoporosis, cardiovascular diseases, cancer, and mortality compared to the general population [5].

The term “bone neoplasms” encompasses all neoplasms originating from bone or various bone tissue components, including primary, secondary, and metastatic neoplasms. Among primary bone neoplasms, benign tumors are more prevalent than malignant ones. Benign bone neoplasms primarily include osteoid osteoma, osteochondroma, and chondroma. Malignant bone neoplasms primarily comprise osteosarcoma, chondrosarcoma, fibrosarcoma of bone, Ewing’s sarcoma, malignant lymphoma, myeloma, and chordoma [6]. Bone neoplasms tend to occur more frequently in the active regions of long bones, namely the metaphysis, such as the distal femur, proximal tibia, and proximal humerus. The main clinical manifestations of bone neoplasms encompass pain, swelling, and functional impairment. Previous investigations have demonstrated an elevated susceptibility to malignancies in individuals with RA [7, 8]. Primary malignant bone tumors constitute approximately 6% of the total malignancies observed in the pediatric population. Osteosarcoma and Ewing’s sarcoma stand as the predominant types within this category, with an annual incidence rate of 8.7% in individuals under the age of 20 [9]. In a retrospective analysis involving nine patients presenting with musculoskeletal pain as their initial symptom, subsequent diagnoses revealed various malignancies, including acute lymphoblastic leukemia, acute myeloid leukemia, lymphoma, neuroblastoma, and Ewing’s sarcoma. Notably, RA was initially diagnosed in four of these cases [10]. Another comprehensive retrospective study spanning 14 years examined 29 pediatric patients with malignancies, encompassing lymphoma, Ewing’s sarcoma, and other malignancies. Surprisingly, initial diagnoses of rheumatoid arthritis were made in 12 of these cases [11]. It is worth noting that individuals with RA exhibit an elevated overall incidence of malignancies, with a 5–10% increased risk compared to the general population. Extensive cohort studies and meta-analyses conducted over the past several decades have consistently demonstrated a significantly heightened risk of lymphoma development in patients with RA, nearly doubling the risk when compared to the general population [12]. And there is an approximately twofold increase in the risk of malignant lymphoma [13]. Malignant lymphoma, also known as primary non-Hodgkin’s lymphoma of the bone, is characterized by the presence of malignant lymphocytes and the formation of swelling lesions in the bone, representing one of the primary malignant bone neoplasms. Although the underlying cause of RA lies in immune system disorder, its primary manifestation is joint inflammation. As RA progresses, progressive arthritis leads to alterations in articular cartilage and eventual joint damage, resulting in impaired function and potential disability for patients. Hence, we hypothesize that RA may augment the risk of bone and articular cartilage neoplasms.

Mendelian randomization (MR) is a data analysis method employed in epidemiological studies to examine causal inferences. The technique utilizes genetic variants, specifically single-nucleotide polymorphisms (SNPs), as instrumental variables (IVs) to estimate the causal relationship between exposure factors and relevant outcomes [14]. Recent advances in genome-wide association analyses (GWAS) have identified numerous genetic variants, often numbering in the hundreds of thousands or even millions, that are associated with disease outcomes. These genetic variants serve as the foundation for conducting MR analyses. By utilizing genetic data, MR enables the assessment of causal effects pertaining to modifiable non-genetic exposures. Compared to conventional observational studies, MR offers the advantage of circumventing confounding factors and reverse causality. Consequently, MR has been widely employed to investigate genetic causality on a large scale [15, 16]. This study employed a two-sample bidirectional MR analysis to investigate the genetic causality between RA and both benign neoplasm of bone and articular cartilage (BNBAC) and malignant neoplasm of bone and articular cartilage (MNBAC).

## Materials and methods

### Data source

The IEU OpenGWAS database (https://gwas.mrcieu.ac.uk/) offers GWAS summary data focused on individuals of European descent who have been diagnosed with RA. The dataset includes both males and females, comprising 14,361 cases and 43,923 controls, with a total of 13,108,512 SNPs analyzed. To assess the genetic correlation of effect sizes across different ethnic groups, the Popcorn method was utilized. For the purpose of identifying independent association signals, a stepwise approximate conditional association analysis was conducted using ancestry-matched linkage disequilibrium (LD) matrices, employing genome-wide complex trait analysis. Imputation of untyped variants in the European individuals within each RA locus was performed using SHAPEIT2 and Minimac3, with the 1KGP imputation reference panel. The published study provides additional details on the data [17]. Concurrently, the Finnish consortium (https://www.finngen.fi/) supplies GWAS summary data for two different conditions, namely BNBAC and MNBAC, encompassing individuals of European descent, both males and females. GWAS summary data for BNBAC and MNBAC are drawn from the general population. The BNBAC dataset consists of 1190 cases and 217,602 controls, yielding 16,380,466 SNPs. Similarly, the MNBAC dataset encompasses 119 cases and 218,673 controls, resulting in 16,380,466 SNPs. The cases within the MNBAC dataset were identified based on the M13 code in the International Classification of Diseases-Tenth Revision (ICD-10). Genotyping for both datasets was carried out using Illumina (Illumina Inc, San Diego) and Affymetrix chip arrays (Thermo Fisher Scientific, Santa Clara, CA, USA). Additional information about the data can be accessed on the FinnGen website. All data employed in this study were obtained from publicly available databases, thus not requiring informed consent or ethical statements. Supplementary Table 1 in this study provides further comprehensive information on the utilized data.

### IVs selection

The identification of causal relationships between exposure and outcome in MR analysis relies on three key assumptions: (1) strong association of IVs with the exposure, (2) independence of IVs from potential confounding factors, and (3) IVs influencing the outcome solely through the exposure. To ensure the robustness of the MR analysis, careful selection of effective IVs is crucial. Initially, we screened SNPs that exhibited a strong correlation with the exposure, setting a stringent threshold of *P* < 5 × 10^−8^ and an *F* statistic > 10. The *F* statistic was calculated using the formula *F* = *R*^2^(*N*-*K*-1)/*K*(1-*R*^2^) [18]. Subsequently, we considered only SNPs with low LD, specifically those with an LD *r*^2^ < 0.001 and a clump distance > 10,000 kb, as valid IVs [19]. Furthermore, to ensure that the IVs were not correlated with the outcome, we applied a correlation threshold of *P* < 5 × 10^−8^. Additionally, we employed the PhenoScanner database to exclude for potential confounding factors. In our study, confounding factors affecting BNBAC and MNBAC, including but not limited to smoking, gender, family genetics, ionizing radiation, harmful chemicals, and viral infections, were considered [20, 21]. Similarly, potential confounding factors affecting RA, such as smoking, obesity, and gender, were taken into account [22, 23]. We also excluded SNPs with palindromic intermediate allele frequencies and incompatible alleles [15]. Finally, in cases where the original SNPs did not match the GWAS summary data of the outcome, we utilized the LDlink online platform to obtain proxy SNPs.

### Statistical analysis

A two-sample MR analysis was conducted, using RA as the exposure and BNBAC and MNBAC as the outcomes. Conversely, BNBAC and MNBAC were used as exposures, and RA was treated as an outcome. The “TwoSampleMR” package in R (version 4.1.2) was employed to perform bidirectional MR analysis. The primary method utilized in the MR analysis was the random-effects inverse variance weighted (IVW) approach. This method incorporates generalized least squares to account for heteroskedastic errors and LD among the SNPs through the variance–covariance matrix [24]. Additionally, alternative MR analysis methods were applied, such as MR Egger, weighted median, simple mode, and weighted mode. The random-effects IVW method combines the Wald ratios of all IVs to estimate causality. Compared to other MR analysis techniques, random-effects IVW exhibits robust statistical properties [25]. To assess the robustness of the causal assessment, a sensitivity analysis of the MR results was performed. Heterogeneity was measured using Cochran’s *Q* statistic for MR-IVW and Rucker’s *Q* statistic for MR Egger [26]. The MR Egger intercept test was used to detect horizontal pleiotropy. The presence of horizontal pleiotropy was also evaluated using the MR pleiotropy residual sum and outlier (MR-PRESSO), which is a powerful method [27]. A *P*-value > 0.05 was considered indicative of the absence of heterogeneity and horizontal pleiotropy. Radial variants of the IVW were used to visually identify outliers [28]. The MR-PRESSO was also employed to statistically detect outliers in the causal assessment [14]. Efforts were made to exclude the influence of outliers on the causal assessment of exposure and outcome. Analyzing the impact of individual SNPs on the causal assessment, a “leave-one-out” analysis was performed for detection [29]. Furthermore, the normal distribution of the MR analyses was examined, and the MR robust adjusted profile score (MR-RAPs) method was employed. A *P*-value > 0.05 was considered indicative of a normal distribution [30]. Finally, the results of the causal assessment were cross-validated using other MR analysis methods, including maximum likelihood, penalized weighted median, and fixed-effects IVW methods.

## Results

### Genetic causality between exposure (RA) and outcomes (BNBAC or MNBAC)

The study utilized a screening process to identify 90 SNPs that demonstrated a strong correlation with RA based on two criteria: meeting the correlation threshold of *P* < 5 × 10^−8^ and having an *F* statistic > 10. The 86 SNPs were selected as alternative IVs after considering the outcome measures of BNBAC or MNBAC. None of the identified SNPs exhibited a significant association with the outcome. However, a confounding SNP (rs9271365) was excluded. Consequently, a total of 85 IVs were ultimately retained for the MR analysis, with one palindromic SNP (rs34536443) being excluded. Further details can be found in Supplementary Tables 2 and 3.

**Fig. 1:**
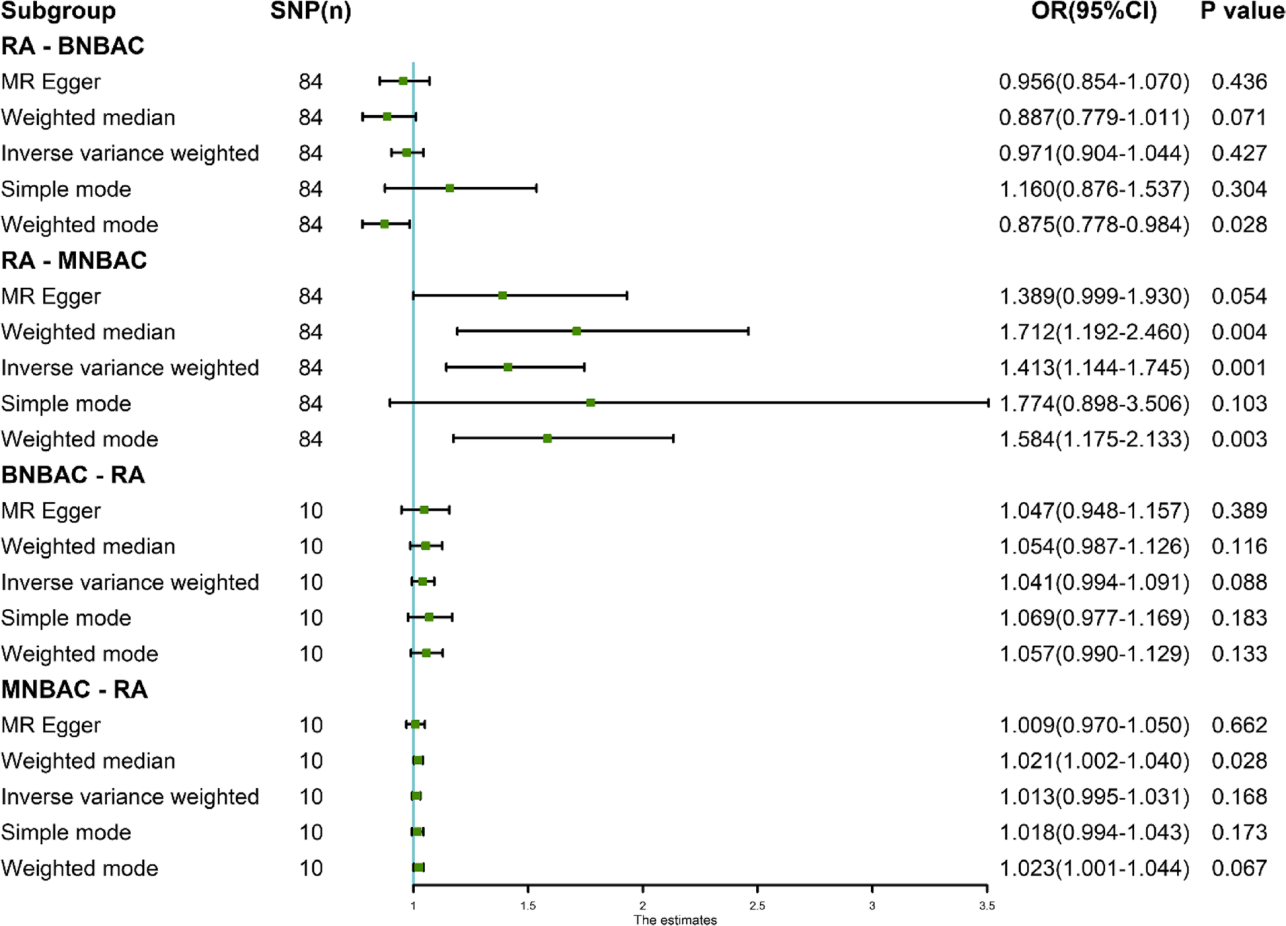
The MR analysis results of RA and BNBAC and MNBAC. The analysis employed five methods, namely random-effects IVW, MR Egger, weighted median, simple mode, and weighted mode

**Fig. 2:**
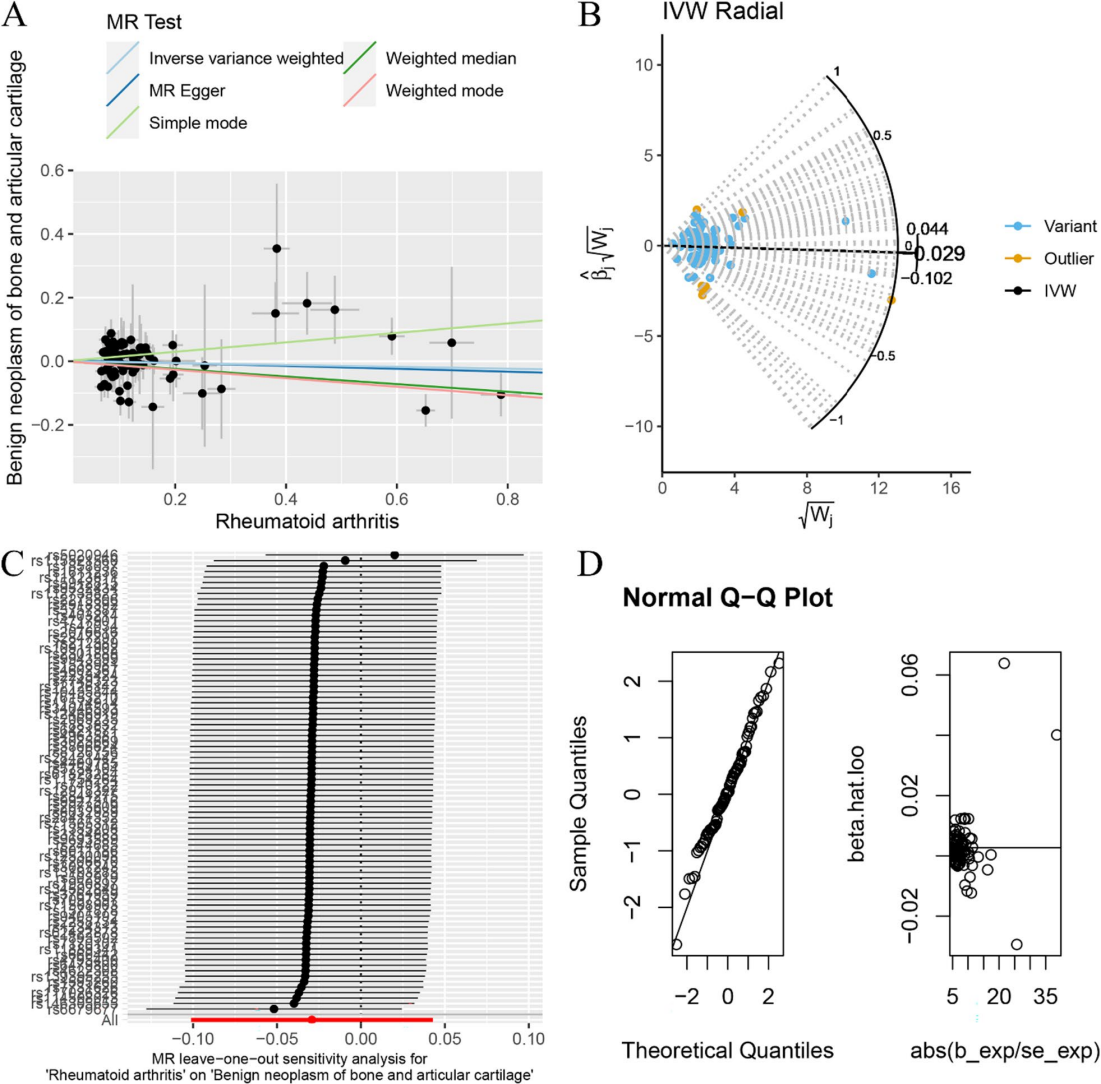
The MR analysis conducted on RA and BNBAC. **A** Scatter plot; **B** MR radial plot; **C** leave-one-out analysis; **D** normal distribution plot

**Fig. 3:**
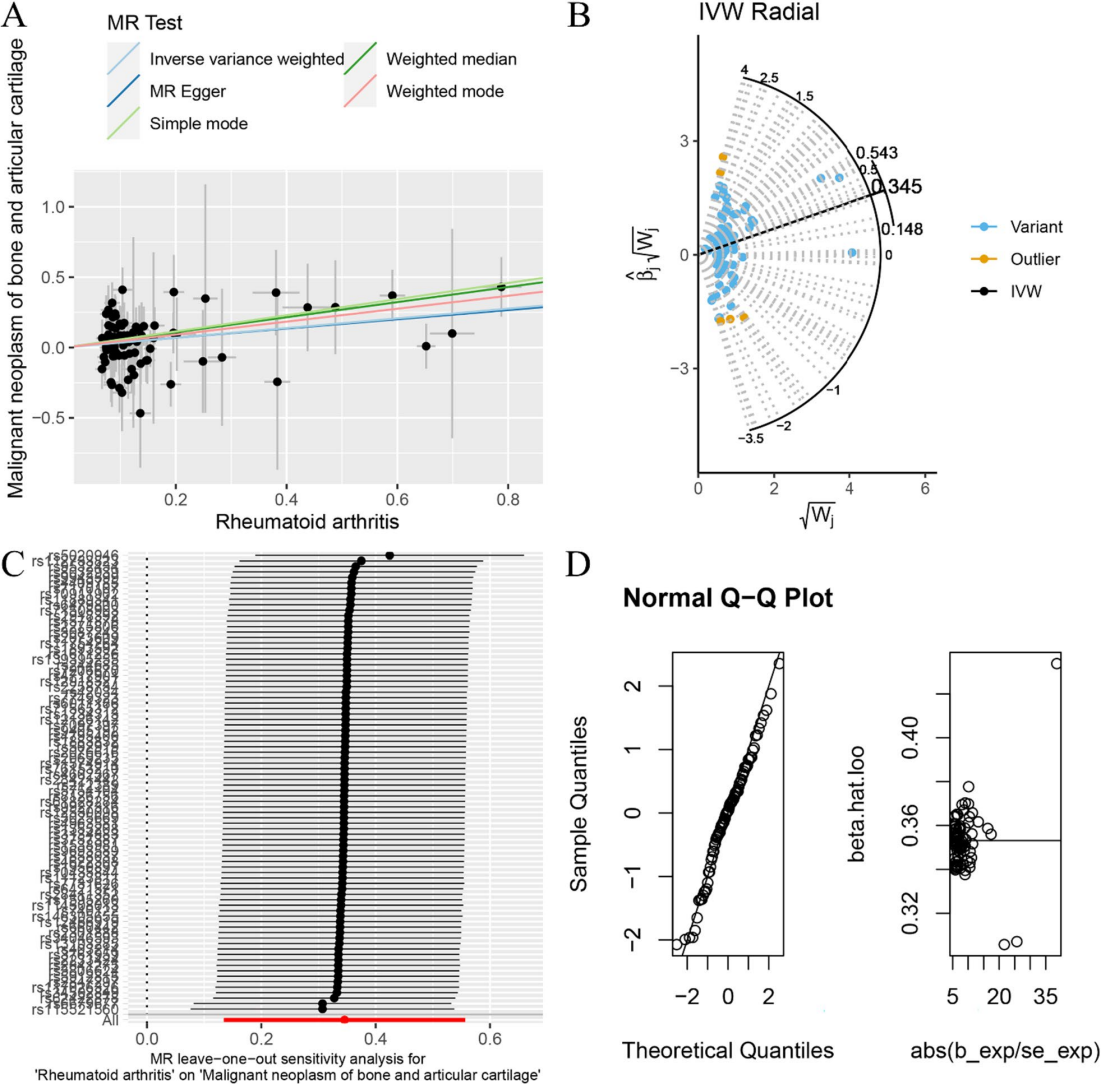
The MR analysis conducted on RA and MNBAC. **A** Scatter plot; **B** MR radial plot; **C** leave-one-out analysis; **D** normal distribution plot

The random-effects IVW method revealed no evidence of genetic causality between RA and BNBAC (*P* = 0.427, odds ratio [OR] 95% confidence interval [CI] = 0.971 [0.904–1.044]). However, a positive genetic causality was observed between RA and MNBAC (*P* = 0.001, OR 95% CI = 1.413 [1.144–1.745]) (Figs. 1, 2A and 3A). Additional causal inference methods including MR Egger, weighted median, simple mode, and weighted mode indicated no genetic causality between RA and BNBAC (*P* > 0.05). Furthermore, MR Egger and simple mode indicated no genetic causality between RA and MNBAC (*P* > 0.05), while weighted median and weighted mode indicated a positive genetic causal relationship (*P* < 0.05 and OR > 1) (Fig. 1). The absence of heterogeneity was demonstrated by the Cochran’s *Q* statistic of MR-IVW and Rucker’s *Q* statistic of MR Egger (*P* > 0.05). The intercept test of MR Egger showed no evidence of horizontal pleiotropy (*P* > 0.05), which was consistent with the results of MR-PRESSO (Table 1). Notably, the radial variants of IVW revealed outliers in the assessment of genetic causality between RA and BNBAC or MNBAC (Figs. 2B and 3B). However, it is important to highlight that the MR-PRESSO indicated the absence of outliers (Table 1). The “leave-one-out” analysis demonstrated that the genetic causality assessment between RA and BNBAC or MNBAC was not influenced by a single SNP (Figs. 2C and 3C). Moreover, the MR-RAPS method revealed a normal distribution in the genetic causality assessment between RA and BNBAC or MNBAC (*P* > 0.05) (Table 1, Figs. 2D and 3D).

**Table 1:** Sensitivity analysis of the MR analysis results of exposures and outcomes

**Exposure**	**Outcome**	**Heterogeneity**		**Pleiotropy**	**MR-PRESSO**		**MR-RAPS**
		**Cochran’s *Q* test (IVW)**	**Rucker’s *Q* test (MR-Egger)**	**Intercept test (MR-Egger)**	**Outlier test**	**Pleiotropy test**	**Normal Distribution**
		***P* value**	***P* value**	***P* value**	**Number**	***P* value**	***P* value**
RA	BNBAC	0.184	0.167	0.720	0	0.152	0.698
RA	MNBAC	0.830	0.809	0.893	0	0.819	0.384
BNBAC	RA	0.500	0.403	0.901	0	0.510	0.973
MNBAC	RA	0.053	0.034	0.847	0	0.338	0.840

*MR* mendelian randomization, *RA* rheumatoid arthritis, *BNBAC* benign neoplasm of bone and articular cartilage, *MNBAC* malignant neoplasm of bone and articular cartilage

Finally, the genetic causal assessment of exposure (RA) and outcomes (BNBAC or MNBAC) was corroborated by employing three distinct methodologies: maximum likelihood, penalized weighted median, and fixed effects IVW. The outcomes of all three approaches indicated that RA exhibited no significant genetic causality with BNBAC (*P* > 0.05). However, a statistically significant positive genetic causality between RA and MNBAC was observed (*P* < 0.05 and OR > 1), as depicted in Fig. 5.

### Genetic causality between exposures (BNBAC or MNBAC) and outcome (RA)

When the selective threshold was set at *P* < 5 × 10^−8^, no SNPs associated with exposure were identified. Consequently, we adjusted the threshold to *P* < 1 × 10^−5^. Following the exclusion of LD, we identified a total of 15 SNPs that exhibited a strong association with BNBAC (*P* < 1 × 10^−5^ and *F* statistic > 10). The 13 SNPs were selected as alternative IVs in collaboration with RA. One RA-related SNP (rs3129774) was excluded from further analysis. Subsequently, we verified that all alternative IVs were not associated with confounding factors. Ultimately, we obtained 12 IVs for examining the genetic causality between BNBAC and RA, including one palindromic SNP (rs6945749) and one SNP with incompatible alleles (rs147722763) (Supplementary Table 4). Similarly, we identified 13 SNPs strongly associated with MNBAC, of which 11 SNPs were found in conjunction with RA. None of these SNPs exhibited associations with RA or confounding factors. Thus, we obtained 11 IVs to investigate the genetic causality between MNBAC and RA, including one SNP with incompatible alleles (rs11042826) (Supplementary Table 5).

**Fig. 4:**
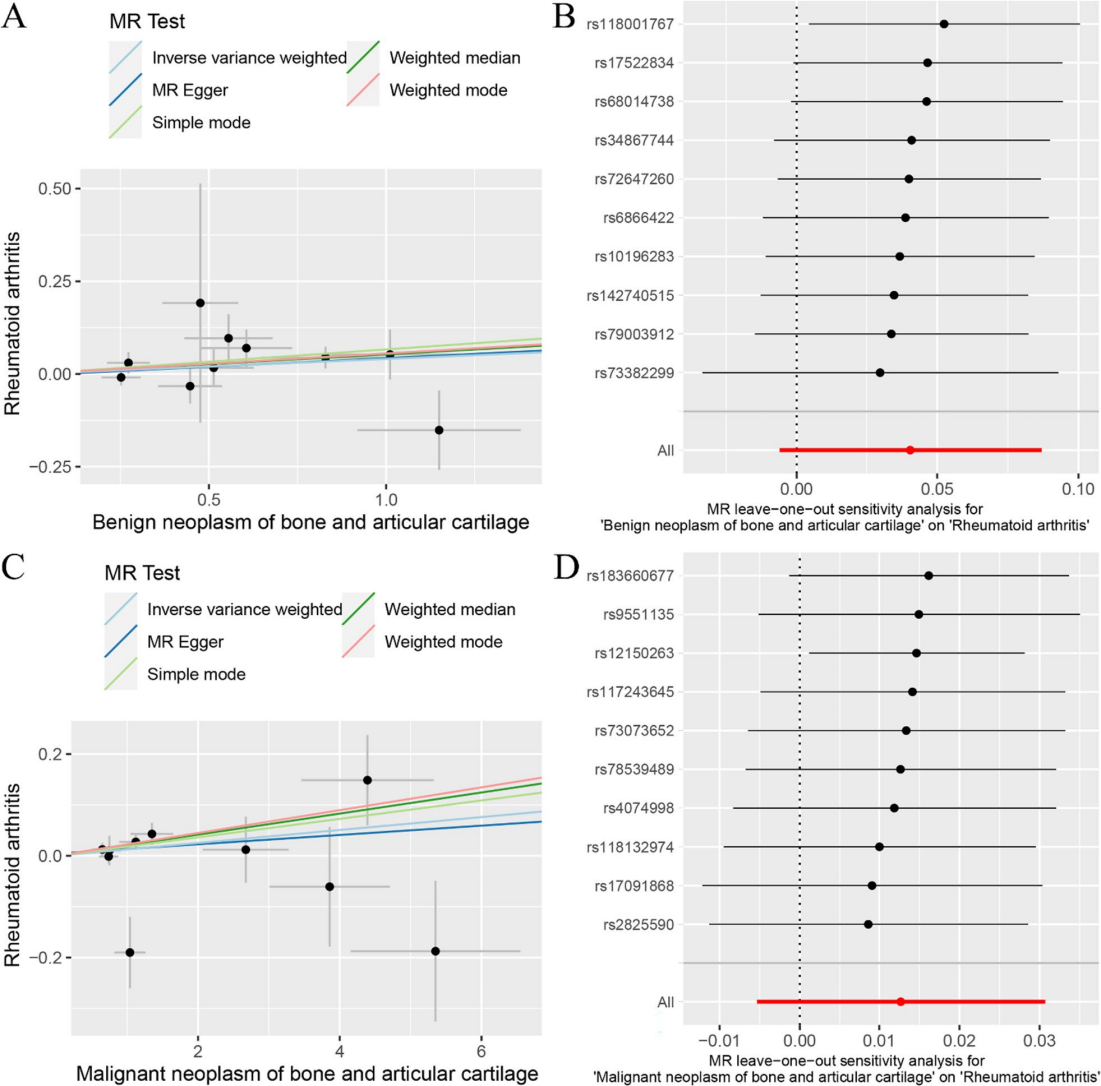
The MR analysis of the exposures (BNBAC and MNBAC) in relation to the outcome (RA). **A** Scatter plot of BNBAC and RA; **B** leave-one-out analysis of BNBAC and RA; **C** scatter plot of MNBAC and RA; **D** leave-one-out analysis of MNBAC and RA

The random-effects IVW method revealed that there was no significant genetic causality between BNBAC and RA (*P* = 0.088, OR 95% [CI] = 1.041 [0.994–1.091]), as well as between MNBAC and RA (*P* = 0.168, OR 95% [CI] = 1.013 [0.995–1.031]) (Figs. 1 and 4A, C). Consistently, the MR Egger, weighted median, simple mode, and weighted mode approaches yielded non-significant results when compared to the random-effects IVW method (*P* > 0.05) (Fig. 1). The assessment of genetic causality between BNBAC and RA showed no evidence of heterogeneity, horizontal pleiotropy, or outliers (Table 1). The Cochran’s *Q* statistic for MR-IVW indicated no significant heterogeneity between MNBAC and RA (*P* > 0.05), while the Rucker’s *Q* statistic for MR Egger showed evidence of heterogeneity (*P* < 0.05). Furthermore, the assessment of genetic causality between MNBAC and RA revealed no indications of horizontal pleiotropy or outliers (Table 1). Additionally, the genetic causality assessment for both BNBAC and MNBAC with RA remained unaffected by individual SNPs (Fig. 4B, D), and the distribution of the genetic effects followed a normal distribution (*P* > 0.05) (Table 1).

**Fig. 5:**
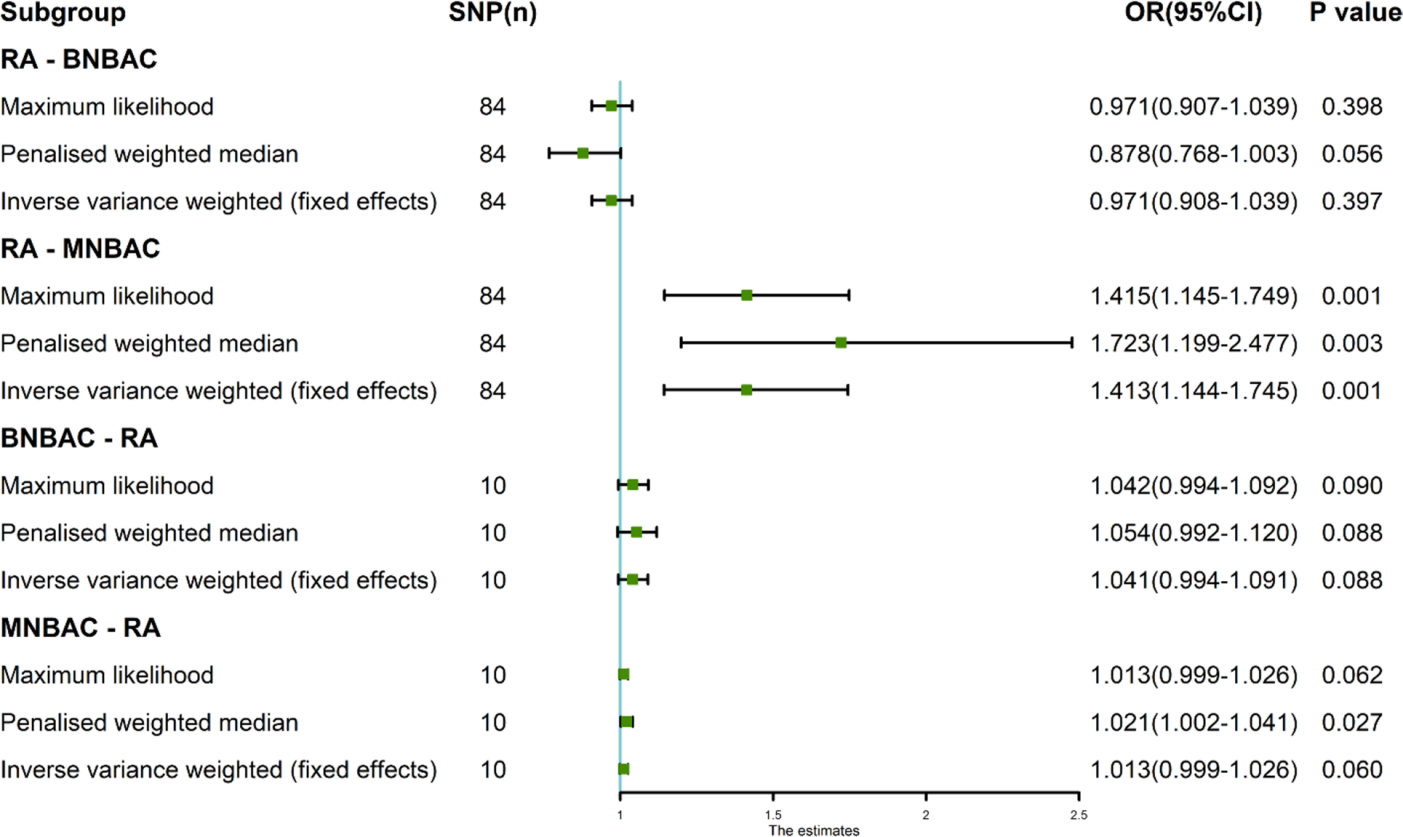
The MR analysis of RA and both BNBAC and MNBAC, utilizing three distinct methods: maximum likelihood, penalized weighted median, and IVW (fixed effects)

The results obtained from three different verification methods, namely maximum likelihood, penalized weighted median, and fixed effects IVW. Specifically, the three analysis reveals that there is no statistically significant genetic causality between BNBAC and RA (*P* > 0.05) (Fig. 5). On the other hand, when examining the relationship between MNBAC and RA, the maximum likelihood and fixed effects IVW methods suggest no genetic causality (*P* > 0.05). However, the penalized weighted median method suggests a positive genetic causality between MNBAC and RA (*P* < 0.05 and OR > 1) (Fig. 5).

## Discussion

In this study, a bidirectional MR analysis was conducted to investigate the relationship between RA and two outcomes, namely BNBAC and MNBAC. Eight MR methods were employed for the analysis. While some of the results obtained from different analysis methods showed inconsistencies, these discrepancies did not substantially impact our findings. Among the methods used, the random effects IVW approach exhibited greater statistical power, making it the primary analytical method employed in this study. Consequently, the findings from our investigation suggest a positive genetic causality between RA and MNBAC, implying that RA serves as a risk factor for MNBAC. However, no evidence of genetic causality was found between RA and BNBAC, RA and MNBAC, or MNBAC and RA.

The immune system plays a pivotal role in the pathogenesis of tumors, exerting multifaceted and intricate influences. Certain types of tumors exhibit a heightened incidence of chronic inflammation and infection, establishing a clear connection between tumor development and these immunological factors. RA, an autoimmune disease characterized by abnormal lymphocyte activity and the generation of autoantibodies against self-antigens, is associated with suppressed immune function. Consequently, dysfunctional immune surveillance and immunosuppression are recognized as risk factors for various types of cancer [31]. Moreover, the utilization of immunosuppressants is linked to an increased susceptibility to tumors. The etiology of tumor risk in RA patients may involve genetic predisposition and gene-environment interactions [7]. Furthermore, the usage of antirheumatic medications is associated with the tumor risk in RA patients [7]. Studies have demonstrated an augmented likelihood of severe infections and a dose-dependent escalation in malignancy risk among RA patients undergoing treatment with anti-tumor necrosis factor (TNF) antibodies [32]. Prolonged use of fostamatinib in patients with RA has also been shown to potentially increase the risk of malignancy [33]. Additionally, chemotherapy, a commonly employed therapeutic approach for cancer, is frequently implicated in immune system impairment and the subsequent elevated risk of autoimmune disorders such as RA. Although the underlying mechanisms remain unclear, it is plausible that genetic and environmental factors contribute to this process [34].

Estrogen metabolites have been implicated in RA and tumor development, and certain estrogen metabolites used for assessing cancer risk also play a significant role in RA. The precise pathway underlying RA-related malignancy remains elusive. One possible mechanism involves the enzymatic or nonenzymatic oxidation of estrogen, resulting in the formation of catechol estrogen metabolites through the semiquinone and quinone redox cycle. This process generates free radicals capable of inducing DNA modifications. These modifications alter the immunogenicity of DNA, triggering various immune responses that lead to elevated levels of tumor and RA antibodies [31]. It is imperative to recognize the variances in estrogen levels across different age groups when examining the potential association between estrogen, RA, and malignancy. As is widely acknowledged, the primary age of RA onset is typically between 30 and 50 years. However, many benign and malignant bone tumors, including osteosarcoma and Ewing’s sarcoma, predominantly manifest during adolescence. In addition, certain bone tumors manifest in middle to late adulthood; for instance, chondrosarcoma is more prevalent in the adult and elderly populations, while malignant lymphoma is commonly diagnosed in individuals aged 40 to 60 years. Additionally, myeloma is more frequently observed in those over the age of 40. Consequently, any estrogen hypothesis positing an association between RA and malignancy must carefully account for patient age, given the age-related variations in estrogen levels. RA patients exhibit a higher incidence of malignancies compared to the general population. RA-associated malignancies include lung cancer, skin cancer, myeloma, non-Hodgkin’s lymphoma and Hodgkin’s disease, lymphoma associated with TNF inhibitors, leukemia, breast cancer, colorectal cancer, and prostate cancer. These malignancies can be attributed to RA medications or the inflammation itself [31]. Disease-modifying anti-rheumatic drugs (DMARDs) currently represent the primary treatment for RA. These drugs modulate the normal immune pathway, influencing the growth and survival of malignant tumors. Although long-term immune dysregulation and inflammatory responses contribute to RA development, they may also increase the risk of cancer. Despite the elevated risk of certain tumors observed in RA patients, the exact mechanism remains unknown due to the complex etiology of the disease. In RA patients, prolonged and sustained activation of the immune system may drive the initiation and progression of cancer, potentially mediated by interleukin-6 (IL-6), which could serve as a common link between RA and cancer [35].

In the context of bone tumor age demographics, a preponderance of cases is observed among adolescents. Benign bone tumors, specifically osteoid osteoma, exhibit a predilection for manifestation in children and adolescents, while osteochondroma primarily afflicts individuals in the adolescent age group. Conversely, malignant bone tumors display distinct patterns: osteosarcoma demonstrates a proclivity towards occurrence in adolescent males, chondrosarcoma tends to affect adults and the elderly, with a male predilection, Ewing’s sarcoma is more frequently diagnosed in children, predominantly males, malignant lymphoma exhibits a peak incidence between the ages of 40 and 60, and myeloma typically presents in males above the age of 40. Although the spectrum of bone tumors is multifaceted, the most prevalent entities are osteosarcoma and Ewing’s sarcoma, both of which predominantly manifest in adolescent males. The general population’s annual incidence of osteosarcoma stands at 2–3 cases per million, although this figure escalates among adolescents, with the highest prevalence occurring within the 15–19 age group, reaching 8–11 cases per million annually. Notably, within this age cohort, osteosarcoma constitutes 15% of all solid extracranial malignancies. Males are afflicted by this condition 1.4 times more frequently than females [36]. Ewing’s sarcoma ranks as the second most prevalent bone tumor among children and adolescents. Data spanning from 1973 to 2004 in the USA documented an incidence rate of 2.93 cases per 1 million. Ewing’s sarcoma exhibits a higher prevalence in the white population, with a slight male predominance [37]. In contrast, RA, characterized as a systemic autoimmune disorder, displays a higher incidence among women. While RA can manifest at any age, its prevalence peaks between the ages of 30 and 50 [2]. The distinction between the demographics of bone tumors, primarily affecting adolescent males, and RA, primarily occurring in middle-aged females, underscores the absence of a discernible age and gender-related association between these two conditions. As our findings suggest, any potential link between RA and bone tumors likely resides at the genetic level.

In this study, our findings suggest a potential association between RA and an increased risk of MNBAC. While no MNBAC cases were observed among the tumor types associated with increased tumor risk in RA patients, the high prevalence of other tumors in this population contributes to the overall conclusion of this investigation. We postulate that the elevated incidence of MNBAC in RA patients may be attributed to the perturbed immune system in these individuals as well as the pharmacological interventions employed for RA treatment. As with any scientific inquiry, it is crucial to acknowledge the inherent limitations of this study. The presence of a limited number of genetic instruments or potential overlap in the sample between exposure and outcome variables may have introduced certain biases. Moreover, the utilization of GWAS summary data exclusively derived from European populations restricts the generalizability of the results to broader demographic groups.

## Conclusion

This study aimed to investigate the genetic causal relationship between RA and both BNBAC and MNBAC using MR analysis. The findings revealed that RA was found to be a significant risk factor for MNBAC. Conversely, RA was not found to increase the risk of BNBAC. Additionally, neither BNBAC nor MNBAC were found to elevate the risk of developing RA. These results contribute novel insights into the incidence of tumors among RA patients. Consequently, clinicians should exercise caution and attentiveness towards the occurrence of MNBAC in patients diagnosed with RA.

### Supplementary Information

**Additional file 1: Supplementary Table 1.** The data used in this study. **Supplementary Table 2.** The instrumental variables used in MR analysis between rheumatoid arthritis and benign neoplasm of bone and articular cartilage. **Supplementary Table 3.** The instrumental variables used in MR analysis between rheumatoid arthritis and malignant neoplasm of bone and articular cartilage. **Supplementary Table 4.** The instrumental variables used in MR analysis between benign neoplasm of bone and articular cartilage and rheumatoid arthritis. **Supplementary Table 5.** The instrumental variables used in MR analysis between malignant neoplasm of bone and articular cartilage and rheumatoid arthritis.

## Data Availability

This study utilized publicly available datasets, which were obtained from the IEU OpenGWAS database (https://gwas.mrcieu.ac.uk/) and the FinnGen consortium (https://www.finngen.fi/).

## Abbreviations

RA: Rheumatoid arthritis
HLA: Human leukocyte antigen
MR: Mendelian randomization
SNP: Single-nucleotide polymorphism
IVs: Instrumental variables
GWAS: Genome-wide association analyses
BNBAC: Benign neoplasm of bone and articular cartilage
MNBAC: Malignant neoplasm of bone and articular cartilage
LD: Linkage disequilibrium
IVW: Inverse variance weighted
MR-PRESSO: MR pleiotropy residual sum and outlier
MR-RAPs: MR robust adjusted profile score
OR: Odds ratio
CI: Confidence interval
TNF: Tumor necrosis factor
DMARDs: Disease-modifying anti-rheumatic drugs
IL-6: Interleukin-6
