# Supplementary material for: Rheumatoid arthritis increases the risk of malignant neoplasm of bone and articular cartilage: a two-sample bidirectional mendelian randomization study
Source: Arthritis Res Ther. 2023 Nov 13;25:219. doi: 10.1186/s13075-023-03205-5 (PMC10642064; doi:10.1186/s13075-023-03205-5)
Supplement: Supplementary file 1 — Additional file 1: Supplementary Table 1. The data used in this study. Supplementary Table 2. The instrumental variables used in MR analysis between rheumatoid arthritis and benign neoplasm of bone and articular cartilage. Supplementary Table 3. The instrumental variables used in MR analysis between rheumatoid arthritis and malignant neoplasm of bone and articular cartilage. Supplementary Table 4. The instrumental variables used in MR analysis between benign neoplasm of bone and articular cartilage and rheumatoid arthritis. Supplementary Table 5. The instrumental variables used in MR analysis between malignant neoplasm of bone and articular cartilage and rheumatoid arthritis. [file 13075_2023_3205_MOESM1_ESM.docx]

**Supplementary Table 1:** The data used in this study.

| **GWAS ID** | **Trait** | **Years** | **Cases** | **Controls** | **SNPs** | **PMID** |
| --- | --- | --- | --- | --- | --- | --- |
| ebi-a-GCST90013534 | Rheumatoid arthritis | 2020 | 14,361 | 43,923 | 13,108,512 | 33310728 |
| finn-b-CD2_BENIGN_BONE_CARTILAGE | Benign neoplasm of bone and articular cartilage | 2021 | 1,190 | 217,602 | 16,380,466 | NA |
| finn-b-C3_BONE_CARTILAGE | Malignant neoplasm of bone and articular cartilage | 2021 | 119 | 218,673 | 16,380,466 | NA |

**Supplementary Table 2:** The instrumental variables used in MR analysis between rheumatoid arthritis and benign neoplasm of bone and articular cartilage.

|  | **SNP** | **beta.exposure** | **se.exposure** | **pval.exposure** | **pval.outcome** | **palindromic** | **F** |
| --- | --- | --- | --- | --- | --- | --- | --- |
| 1 | rs10435844 | -0.0784 | 0.0121 | 9.73E-11 | 0.6022 | FALSE | 41.98039 |
| 2 | rs10911902 | -0.0847 | 0.0152 | 2.36E-08 | 0.3918 | FALSE | 31.05022 |
| 3 | rs11123811 | -0.0995 | 0.0114 | 2.01E-18 | 0.02319 | FALSE | 76.17659 |
| 4 | rs112733823 | 0.191 | 0.0188 | 3.82E-24 | 0.2845 | FALSE | 103.2134 |
| 5 | rs114508013 | 0.488 | 0.0441 | 1.82E-28 | 0.1298 | FALSE | 122.4468 |
| 6 | rs115521560 | 0.7878 | 0.0364 | 1.29E-103 | 0.1211 | FALSE | 468.3972 |
| 7 | rs11574914 | 0.1153 | 0.0149 | 9.92E-15 | 0.7674 | FALSE | 59.87854 |
| 8 | rs117026326 | 0.381 | 0.0424 | 2.45E-19 | 0.1246 | FALSE | 80.74271 |
| 9 | rs11754264 | -0.1359 | 0.0193 | 1.88E-12 | 0.866 | FALSE | 49.58033 |
| 10 | rs11889341 | 0.1466 | 0.0129 | 4.32E-30 | 0.3844 | FALSE | 129.1438 |
| 11 | rs12126142 | -0.0751 | 0.0116 | 1.01E-10 | 0.552 | FALSE | 41.91302 |
| 12 | rs1234313 | 0.0797 | 0.0133 | 1.90E-09 | 0.1979 | FALSE | 35.9086 |
| 13 | rs12466919 | 0.1025 | 0.0152 | 1.59E-11 | 0.7692 | FALSE | 45.47217 |
| 14 | rs12530098 | 0.1382 | 0.0204 | 1.35E-11 | 0.571 | FALSE | 45.89241 |
| 15 | rs12918327 | 0.0867 | 0.0157 | 3.04E-08 | 0.8462 | FALSE | 30.49467 |
| 16 | rs13103285 | 0.0989 | 0.0131 | 4.29E-14 | 0.6498 | FALSE | 56.99478 |
| 17 | rs1355208 | 0.0818 | 0.0119 | 6.77E-12 | 0.6509 | FALSE | 47.24956 |
| 18 | rs139395255 | 0.3833 | 0.0235 | 8.68E-60 | 0.08257 | FALSE | 266.0278 |
| 19 | rs146305655 | -0.4379 | 0.0452 | 3.29E-22 | 0.06458 | FALSE | 93.85515 |
| 20 | rs1538981 | 0.0671 | 0.0114 | 4.42E-09 | 0.4619 | FALSE | 34.64339 |
| 21 | rs1571878 | -0.1539 | 0.0116 | 4.13E-40 | 0.7218 | FALSE | 176.0137 |
| 22 | rs1595260 | 0.0845 | 0.0126 | 2.29E-11 | 0.04744 | FALSE | 44.97358 |
| 23 | rs1611236 | -0.1165 | 0.0131 | 4.54E-19 | 0.01343 | FALSE | 79.08504 |
| 24 | rs1858037 | -0.1012 | 0.0131 | 1.14E-14 | 0.006226 | FALSE | 59.67653 |
| 25 | rs1883832 | 0.1052 | 0.0127 | 1.13E-16 | 0.8116 | FALSE | 68.61343 |
| 26 | rs1893592 | -0.0976 | 0.0132 | 1.48E-13 | 0.2339 | FALSE | 54.66846 |
| 27 | rs1950897 | 0.1069 | 0.0144 | 1.02E-13 | 0.6303 | FALSE | 55.10811 |
| 28 | rs2069235 | 0.1296 | 0.014 | 1.69E-20 | 0.8163 | FALSE | 85.69175 |
| 29 | rs2073609 | 0.1029 | 0.0182 | 1.47E-08 | 0.5653 | FALSE | 31.96488 |
| 30 | rs2076616 | -0.0885 | 0.0135 | 6.20E-11 | 0.3809 | FALSE | 42.97383 |
| 31 | rs212389 | 0.1058 | 0.0147 | 6.66E-13 | 0.5067 | FALSE | 51.79905 |
| 32 | rs2233424 | 0.1964 | 0.0187 | 6.49E-26 | 0.614899 | FALSE | 110.3024 |
| 33 | rs2258734 | -0.0921 | 0.0123 | 6.04E-14 | 0.3314 | FALSE | 56.0653 |
| 34 | rs2275806 | -0.0725 | 0.0122 | 2.51E-09 | 0.082871 | FALSE | 35.31356 |
| 35 | rs2301888 | -0.1282 | 0.0121 | 3.75E-26 | 0.5915 | FALSE | 112.251 |
| 36 | rs244685 | -0.089 | 0.0144 | 6.04E-10 | 0.6088 | FALSE | 38.19796 |
| 37 | rs28411352 | 0.0914 | 0.0136 | 1.66E-11 | 0.701799 | FALSE | 45.16476 |
| 38 | rs2841275 | 0.1617 | 0.0179 | 1.71E-19 | 0.967 | FALSE | 81.60168 |
| 39 | rs28421442 | -0.1234 | 0.0214 | 7.86E-09 | 0.9056 | FALSE | 33.24971 |
| 40 | rs2847297 | 0.0903 | 0.0119 | 2.65E-14 | 0.4563 | FALSE | 57.57934 |
| 41 | rs2918392 | 0.0668 | 0.0122 | 4.62E-08 | 0.077831 | FALSE | 29.97908 |
| 42 | rs3025669 | -0.2534 | 0.0222 | 2.91E-30 | 0.9565 | FALSE | 130.2844 |
| 43 | rs3087243 | -0.1261 | 0.0124 | 3.32E-24 | 0.7462 | FALSE | 103.4122 |
| 44 | rs3134883 | 0.0991 | 0.0125 | 1.98E-15 | 0.7059 | FALSE | 62.85103 |
| 45 | rs34046593 | 0.1422 | 0.017 | 7.17E-17 | 0.790901 | FALSE | 69.9659 |
| 46 | rs34502849 | -0.0851 | 0.014 | 1.07E-09 | 0.495 | FALSE | 36.94776 |
| 47 | rs34536443 | -0.3801 | 0.0474 | 1.08E-15 | 0.8368 | TRUE | 64.30195 |
| 48 | rs3757387 | 0.1236 | 0.0137 | 1.87E-19 | 0.4069 | FALSE | 81.39185 |
| 49 | rs3761959 | 0.0744 | 0.0115 | 9.64E-11 | 0.4588 | FALSE | 41.85384 |
| 50 | rs3806624 | 0.0863 | 0.0131 | 3.94E-11 | 0.9447 | FALSE | 43.39744 |
| 51 | rs403214 | -0.0914 | 0.0146 | 3.96E-10 | 0.3142 | FALSE | 39.18969 |
| 52 | rs42034 | 0.0871 | 0.0153 | 1.28E-08 | 0.3129 | FALSE | 32.40698 |
| 53 | rs4409785 | 0.0982 | 0.017 | 7.85E-09 | 0.9711 | FALSE | 33.36647 |
| 54 | rs4602367 | 0.075 | 0.0117 | 1.76E-10 | 0.521 | FALSE | 41.08998 |
| 55 | rs4622308 | 0.0878 | 0.0125 | 2.21E-12 | 0.1443 | FALSE | 49.33488 |
| 56 | rs4717901 | 0.249 | 0.0349 | 9.52E-13 | 0.3751 | FALSE | 50.90178 |
| 57 | rs4795400 | 0.0743 | 0.012 | 5.86E-10 | 0.1016 | FALSE | 38.33542 |
| 58 | rs4963581 | 0.0856 | 0.0156 | 3.75E-08 | 0.9335 | FALSE | 30.10811 |
| 59 | rs5020946 | 0.6519 | 0.0169 | 1.00E-200 | 0.002618 | FALSE | 1487.9 |
| 60 | rs502919 | 0.0829 | 0.0134 | 6.17E-10 | 0.4247 | FALSE | 38.2723 |
| 61 | rs5754104 | 0.0891 | 0.0139 | 1.36E-10 | 0.9701 | FALSE | 41.08761 |
| 62 | rs5912815 | -0.0787 | 0.0134 | 4.79E-09 | 0.02581 | FALSE | 34.49252 |
| 63 | rs6011186 | -0.1074 | 0.0171 | 3.19E-10 | 0.4643 | FALSE | 39.44586 |
| 64 | rs61828284 | -0.2018 | 0.0348 | 6.33E-09 | 0.9917 | FALSE | 33.62551 |
| 65 | rs62422878 | 0.1037 | 0.0176 | 3.57E-09 | 0.2385 | FALSE | 34.71501 |
| 66 | rs6421571 | 0.134 | 0.0178 | 5.57E-14 | 0.903 | FALSE | 56.67019 |
| 67 | rs6479800 | 0.1202 | 0.0181 | 3.01E-11 | 0.2001 | FALSE | 44.09983 |
| 68 | rs660442 | -0.1067 | 0.0175 | 1.11E-09 | 0.2239 | FALSE | 37.17388 |
| 69 | rs6679677 | 0.591 | 0.023 | 1.41E-145 | 0.1776 | FALSE | 660.2439 |
| 70 | rs7097397 | -0.0847 | 0.012 | 1.42E-12 | 0.4615 | FALSE | 49.81836 |
| 71 | rs71508903 | 0.1487 | 0.0143 | 3.13E-25 | 0.613901 | FALSE | 108.1272 |
| 72 | rs71565312 | 0.699 | 0.0402 | 1.11E-67 | 0.8079 | FALSE | 302.3342 |
| 73 | rs7170107 | 0.1366 | 0.0158 | 6.11E-18 | 0.3714 | FALSE | 74.74331 |
| 74 | rs7206670 | 0.0701 | 0.0119 | 4.14E-09 | 0.5077 | FALSE | 34.69982 |
| 75 | rs740122 | -0.0782 | 0.0134 | 5.37E-09 | 0.8432 | FALSE | 34.05564 |
| 76 | rs76153210 | 0.1597 | 0.0205 | 6.83E-15 | 0.4643 | FALSE | 60.68582 |
| 77 | rs7731626 | -0.1956 | 0.0184 | 1.94E-26 | 0.2731 | FALSE | 113.0023 |
| 78 | rs7749323 | 0.2835 | 0.0253 | 3.47E-29 | 0.578 | FALSE | 125.5597 |
| 79 | rs8032939 | 0.1244 | 0.0123 | 4.47E-24 | 0.8485 | FALSE | 102.2859 |
| 80 | rs8126756 | -0.0823 | 0.0137 | 1.81E-09 | 0.9687 | FALSE | 36.08641 |
| 81 | rs9271365 | 0.4888 | 0.0128 | 1.00E-200 | 0.7955 | FALSE | 1458.235 |
| 82 | rs9405192 | -0.089 | 0.0137 | 9.26E-11 | 0.412 | FALSE | 42.20112 |
| 83 | rs9532434 | 0.114 | 0.0126 | 1.94E-19 | 0.075501 | FALSE | 81.8566 |
| 84 | rs9693589 | 0.1127 | 0.0128 | 1.50E-18 | 0.6598 | FALSE | 77.51986 |
| 85 | rs9927316 | 0.0906 | 0.0136 | 2.30E-11 | 0.7486 | FALSE | 44.37759 |
| 86 | rs9943599 | 0.083 | 0.0131 | 2.70E-10 | 0.4596 | FALSE | 40.14197 |
| Confounding | |  | | | | | |
| 1 | rs9271365 | Illnesses of father: lung cancer | | | | | |

**Supplementary Table 3:** The instrumental variables used in MR analysis between rheumatoid arthritis and malignant neoplasm of bone and articular cartilage.

|  | **SNP** | **beta.exposure** | **se.exposure** | **pval.exposure** | **pval.outcome** | **palindromic** | **F** |
| --- | --- | --- | --- | --- | --- | --- | --- |
| 1 | rs10435844 | -0.0784 | 0.0121 | 9.73E-11 | 0.4855 | FALSE | 41.98039 |
| 2 | rs10911902 | -0.0847 | 0.0152 | 2.36E-08 | 0.091401 | FALSE | 31.05022 |
| 3 | rs11123811 | -0.0995 | 0.0114 | 2.01E-18 | 0.5115 | FALSE | 76.17659 |
| 4 | rs112733823 | 0.191 | 0.0188 | 3.82E-24 | 0.09904 | FALSE | 103.2134 |
| 5 | rs114508013 | 0.488 | 0.0441 | 1.82E-28 | 0.3907 | FALSE | 122.4468 |
| 6 | rs115521560 | 0.7878 | 0.0364 | 1.29E-103 | 0.04183 | FALSE | 468.3972 |
| 7 | rs11574914 | 0.1153 | 0.0149 | 9.92E-15 | 0.7861 | FALSE | 59.87854 |
| 8 | rs117026326 | 0.381 | 0.0424 | 2.45E-19 | 0.1986 | FALSE | 80.74271 |
| 9 | rs11754264 | -0.1359 | 0.0193 | 1.88E-12 | 0.2279 | FALSE | 49.58033 |
| 10 | rs11889341 | 0.1466 | 0.0129 | 4.32E-30 | 0.549201 | FALSE | 129.1438 |
| 11 | rs12126142 | -0.0751 | 0.0116 | 1.01E-10 | 0.8966 | FALSE | 41.91302 |
| 12 | rs1234313 | 0.0797 | 0.0133 | 1.90E-09 | 0.919 | FALSE | 35.9086 |
| 13 | rs12466919 | 0.1025 | 0.0152 | 1.59E-11 | 0.2554 | FALSE | 45.47217 |
| 14 | rs12530098 | 0.1382 | 0.0204 | 1.35E-11 | 0.719999 | FALSE | 45.89241 |
| 15 | rs12918327 | 0.0867 | 0.0157 | 3.04E-08 | 0.6848 | FALSE | 30.49467 |
| 16 | rs13103285 | 0.0989 | 0.0131 | 4.29E-14 | 0.1959 | FALSE | 56.99478 |
| 17 | rs1355208 | 0.0818 | 0.0119 | 6.77E-12 | 0.6979 | FALSE | 47.24956 |
| 18 | rs139395255 | 0.3833 | 0.0235 | 8.68E-60 | 0.696101 | FALSE | 266.0278 |
| 19 | rs146305655 | -0.4379 | 0.0452 | 3.29E-22 | 0.3594 | FALSE | 93.85515 |
| 20 | rs1538981 | 0.0671 | 0.0114 | 4.42E-09 | 0.615 | FALSE | 34.64339 |
| 21 | rs1571878 | -0.1539 | 0.0116 | 4.13E-40 | 0.9502 | FALSE | 176.0137 |
| 22 | rs1595260 | 0.0845 | 0.0126 | 2.29E-11 | 0.2904 | FALSE | 44.97358 |
| 23 | rs1611236 | -0.1165 | 0.0131 | 4.54E-19 | 0.7879 | FALSE | 79.08504 |
| 24 | rs1858037 | -0.1012 | 0.0131 | 1.14E-14 | 0.5708 | FALSE | 59.67653 |
| 25 | rs1883832 | 0.1052 | 0.0127 | 1.13E-16 | 0.9167 | FALSE | 68.61343 |
| 26 | rs1893592 | -0.0976 | 0.0132 | 1.48E-13 | 0.6849 | FALSE | 54.66846 |
| 27 | rs1950897 | 0.1069 | 0.0144 | 1.02E-13 | 0.543 | FALSE | 55.10811 |
| 28 | rs2069235 | 0.1296 | 0.014 | 1.69E-20 | 0.7648 | FALSE | 85.69175 |
| 29 | rs2073609 | 0.1029 | 0.0182 | 1.47E-08 | 0.2433 | FALSE | 31.96488 |
| 30 | rs2076616 | -0.0885 | 0.0135 | 6.20E-11 | 0.8911 | FALSE | 42.97383 |
| 31 | rs212389 | 0.1058 | 0.0147 | 6.66E-13 | 0.770699 | FALSE | 51.79905 |
| 32 | rs2233424 | 0.1964 | 0.0187 | 6.49E-26 | 0.1358 | FALSE | 110.3024 |
| 33 | rs2258734 | -0.0921 | 0.0123 | 6.04E-14 | 0.788099 | FALSE | 56.0653 |
| 34 | rs2275806 | -0.0725 | 0.0122 | 2.51E-09 | 0.4274 | FALSE | 35.31356 |
| 35 | rs2301888 | -0.1282 | 0.0121 | 3.75E-26 | 0.2867 | FALSE | 112.251 |
| 36 | rs244685 | -0.089 | 0.0144 | 6.04E-10 | 0.684101 | FALSE | 38.19796 |
| 37 | rs28411352 | 0.0914 | 0.0136 | 1.66E-11 | 0.3432 | FALSE | 45.16476 |
| 38 | rs2841275 | 0.1617 | 0.0179 | 1.71E-19 | 0.2377 | FALSE | 81.60168 |
| 39 | rs28421442 | -0.1234 | 0.0214 | 7.86E-09 | 0.8743 | FALSE | 33.24971 |
| 40 | rs2847297 | 0.0903 | 0.0119 | 2.65E-14 | 0.07647 | FALSE | 57.57934 |
| 41 | rs2918392 | 0.0668 | 0.0122 | 4.62E-08 | 0.2844 | FALSE | 29.97908 |
| 42 | rs3025669 | -0.2534 | 0.0222 | 2.91E-30 | 0.667999 | FALSE | 130.2844 |
| 43 | rs3087243 | -0.1261 | 0.0124 | 3.32E-24 | 0.7892 | FALSE | 103.4122 |
| 44 | rs3134883 | 0.0991 | 0.0125 | 1.98E-15 | 0.6541 | FALSE | 62.85103 |
| 45 | rs34046593 | 0.1422 | 0.017 | 7.17E-17 | 0.279 | FALSE | 69.9659 |
| 46 | rs34502849 | -0.0851 | 0.014 | 1.07E-09 | 0.03004 | FALSE | 36.94776 |
| 47 | rs34536443 | -0.3801 | 0.0474 | 1.08E-15 | 0.3884 | TRUE | 64.30195 |
| 48 | rs3757387 | 0.1236 | 0.0137 | 1.87E-19 | 0.62 | FALSE | 81.39185 |
| 49 | rs3761959 | 0.0744 | 0.0115 | 9.64E-11 | 0.06917 | FALSE | 41.85384 |
| 50 | rs3806624 | 0.0863 | 0.0131 | 3.94E-11 | 0.09803 | FALSE | 43.39744 |
| 51 | rs403214 | -0.0914 | 0.0146 | 3.96E-10 | 0.1098 | FALSE | 39.18969 |
| 52 | rs42034 | 0.0871 | 0.0153 | 1.28E-08 | 0.8667 | FALSE | 32.40698 |
| 53 | rs4409785 | 0.0982 | 0.017 | 7.85E-09 | 0.098141 | FALSE | 33.36647 |
| 54 | rs4602367 | 0.075 | 0.0117 | 1.76E-10 | 0.8352 | FALSE | 41.08998 |
| 55 | rs4622308 | 0.0878 | 0.0125 | 2.21E-12 | 0.5637 | FALSE | 49.33488 |
| 56 | rs4717901 | 0.249 | 0.0349 | 9.52E-13 | 0.7871 | FALSE | 50.90178 |
| 57 | rs4795400 | 0.0743 | 0.012 | 5.86E-10 | 0.9983 | FALSE | 38.33542 |
| 58 | rs4963581 | 0.0856 | 0.0156 | 3.75E-08 | 0.701601 | FALSE | 30.10811 |
| 59 | rs5020946 | 0.6519 | 0.0169 | 1.00E-200 | 0.9524 | FALSE | 1487.9 |
| 60 | rs502919 | 0.0829 | 0.0134 | 6.17E-10 | 0.9947 | FALSE | 38.2723 |
| 61 | rs5754104 | 0.0891 | 0.0139 | 1.36E-10 | 0.788301 | FALSE | 41.08761 |
| 62 | rs5912815 | -0.0787 | 0.0134 | 4.79E-09 | 0.1302 | FALSE | 34.49252 |
| 63 | rs6011186 | -0.1074 | 0.0171 | 3.19E-10 | 0.8057 | FALSE | 39.44586 |
| 64 | rs61828284 | -0.2018 | 0.0348 | 6.33E-09 | 0.716 | FALSE | 33.62551 |
| 65 | rs62422878 | 0.1037 | 0.0176 | 3.57E-09 | 0.00974 | FALSE | 34.71501 |
| 66 | rs6421571 | 0.134 | 0.0178 | 5.57E-14 | 0.4592 | FALSE | 56.67019 |
| 67 | rs6479800 | 0.1202 | 0.0181 | 3.01E-11 | 0.348 | FALSE | 44.09983 |
| 68 | rs660442 | -0.1067 | 0.0175 | 1.11E-09 | 0.2354 | FALSE | 37.17388 |
| 69 | rs6679677 | 0.591 | 0.023 | 1.41E-145 | 0.04333 | FALSE | 660.2439 |
| 70 | rs7097397 | -0.0847 | 0.012 | 1.42E-12 | 0.9783 | FALSE | 49.81836 |
| 71 | rs71508903 | 0.1487 | 0.0143 | 3.13E-25 | 0.590799 | FALSE | 108.1272 |
| 72 | rs71565312 | 0.699 | 0.0402 | 1.11E-67 | 0.8934 | FALSE | 302.3342 |
| 73 | rs7170107 | 0.1366 | 0.0158 | 6.11E-18 | 0.4387 | FALSE | 74.74331 |
| 74 | rs7206670 | 0.0701 | 0.0119 | 4.14E-09 | 0.611699 | FALSE | 34.69982 |
| 75 | rs740122 | -0.0782 | 0.0134 | 5.37E-09 | 0.129 | FALSE | 34.05564 |
| 76 | rs76153210 | 0.1597 | 0.0205 | 6.83E-15 | 0.9119 | FALSE | 60.68582 |
| 77 | rs7731626 | -0.1956 | 0.0184 | 1.94E-26 | 0.4737 | FALSE | 113.0023 |
| 78 | rs7749323 | 0.2835 | 0.0253 | 3.47E-29 | 0.8861 | FALSE | 125.5597 |
| 79 | rs8032939 | 0.1244 | 0.0123 | 4.47E-24 | 0.1771 | FALSE | 102.2859 |
| 80 | rs8126756 | -0.0823 | 0.0137 | 1.81E-09 | 0.749601 | FALSE | 36.08641 |
| 81 | rs9271365 | 0.4888 | 0.0128 | 1.00E-200 | 0.5528 | FALSE | 1458.235 |
| 82 | rs9405192 | -0.089 | 0.0137 | 9.26E-11 | 0.9856 | FALSE | 42.20112 |
| 83 | rs9532434 | 0.114 | 0.0126 | 1.94E-19 | 0.08952 | FALSE | 81.8566 |
| 84 | rs9693589 | 0.1127 | 0.0128 | 1.50E-18 | 0.611499 | FALSE | 77.51986 |
| 85 | rs9927316 | 0.0906 | 0.0136 | 2.30E-11 | 0.7438 | FALSE | 44.37759 |
| 86 | rs9943599 | 0.083 | 0.0131 | 2.70E-10 | 0.078921 | FALSE | 40.14197 |
| Confounding | |  | | | | | |
| 1 | rs9271365 | Illnesses of father: lung cancer | | | | | |

**Supplementary Table 4:** The instrumental variables used in MR analysis between benign neoplasm of bone and articular cartilage and rheumatoid arthritis.

|  | **SNP** | **beta.exposure** | **se.exposure** | **pval.exposure** | **pval.outcome** | **palindromic** | **F** |
| --- | --- | --- | --- | --- | --- | --- | --- |
| 1 | rs10196283 | 0.2728 | 0.0602 | 5.81E-06 | 0.2969 | FALSE | 20.53486 |
| 2 | rs118001767 | 1.1496 | 0.2304 | 6.04E-07 | 0.1569 | FALSE | 24.89571 |
| 3 | rs142740515 | 0.5553 | 0.1247 | 8.46E-06 | 0.1379 | FALSE | 19.82981 |
| 4 | rs147722763 | 3.6491 | 0.8205 | 8.69E-06 | 0.06086 | FALSE | 19.77928 |
| 5 | rs17522834 | 0.4468 | 0.0903 | 7.58E-07 | 0.4846 | FALSE | 24.482 |
| 6 | rs3129774 | -0.1959 | 0.0434 | 6.38E-06 | 8.00E-169 | FALSE | 20.37443 |
| 7 | rs34867744 | 0.5134 | 0.1127 | 5.24E-06 | 0.7361 | FALSE | 20.752 |
| 8 | rs68014738 | 0.2521 | 0.0553 | 5.14E-06 | 0.6831 | FALSE | 20.7822 |
| 9 | rs6866422 | 1.0112 | 0.2268 | 8.23E-06 | 0.4344 | FALSE | 19.87852 |
| 10 | rs6945749 | 0.189 | 0.0419 | 6.49E-06 | 0.01514 | TRUE | 20.34659 |
| 11 | rs72647260 | 0.4756 | 0.1069 | 8.72E-06 | 0.5526 | FALSE | 19.79358 |
| 12 | rs73382299 | 0.8289 | 0.1818 | 5.12E-06 | 0.131 | FALSE | 20.78799 |
| 13 | rs79003912 | 0.6056 | 0.1292 | 2.78E-06 | 0.1641 | FALSE | 21.97064 |

**Supplementary Table 5:** The instrumental variables used in MR analysis between malignant neoplasm of bone and articular cartilage and rheumatoid arthritis.

|  | **SNP** | **beta.exposure** | **se.exposure** | **pval.exposure** | **pval.outcome** | **palindromic** | **F** |
| --- | --- | --- | --- | --- | --- | --- | --- |
| 1 | rs11042826 | 8.8629 | 1.9566 | 5.90E-06 | 0.8244 | FALSE | 20.51841 |
| 2 | rs117243645 | 3.8579 | 0.851 | 5.80E-06 | 0.6045 | FALSE | 20.55128 |
| 3 | rs118132974 | 4.3915 | 0.9331 | 2.52E-06 | 0.09351 | FALSE | 22.14958 |
| 4 | rs12150263 | 1.0397 | 0.2184 | 1.94E-06 | 0.006697 | FALSE | 22.66245 |
| 5 | rs17091868 | 1.1224 | 0.2328 | 1.43E-06 | 0.078781 | FALSE | 23.24478 |
| 6 | rs183660677 | 5.3517 | 1.1965 | 7.72E-06 | 0.1748 | FALSE | 20.00572 |
| 7 | rs2825590 | -1.3504 | 0.2997 | 6.62E-06 | 0.04852 | FALSE | 20.3024 |
| 8 | rs4074998 | 0.65 | 0.1377 | 2.34E-06 | 0.3441 | FALSE | 22.28204 |
| 9 | rs73073652 | 2.6747 | 0.6042 | 9.57E-06 | 0.8538 | FALSE | 19.59678 |
| 10 | rs78539489 | 0.7494 | 0.1607 | 3.09E-06 | 0.723701 | FALSE | 21.74661 |
| 11 | rs9551135 | 0.7397 | 0.1373 | 7.21E-08 | 0.9195 | FALSE | 29.0246 |
